# Smartband Use During Enhanced Recovery After Surgery Facilitates Inpatient Recuperation Following Minimally Invasive Colorectal Surgery

**DOI:** 10.3389/fsurg.2020.608950

**Published:** 2021-01-20

**Authors:** Tzu-Chieh Yin, Ching-Wen Huang, Hsiang-Lin Tsai, Wei-Chih Su, Cheng-Jen Ma, Tsung-Kun Chang, Jaw-Yuan Wang

**Affiliations:** ^1^Division of General and Digestive Surgery, Department of Surgery, Kaohsiung Medical University Hospital, Kaohsiung Medical University, Kaohsiung, Taiwan; ^2^Department of Surgery, Kaohsiung Municipal Tatung Hospital, Kaohsiung Medical University, Kaohsiung, Taiwan; ^3^Division of Colorectal Surgery, Department of Surgery, Kaohsiung Medical University Hospital, Kaohsiung Medical University, Kaohsiung, Taiwan; ^4^Department of Surgery, Faculty of Medicine, College of Medicine, Kaohsiung Medical University, Kaohsiung, Taiwan; ^5^Graduate Institute of Clinical Medicine, College of Medicine, Kaohsiung Medical University, Kaohsiung, Taiwan; ^6^Graduate Institute of Medicine, College of Medicine, Kaohsiung Medical University, Kaohsiung, Taiwan; ^7^Center for Cancer Research, Kaohsiung Medical University, Kaohsiung, Taiwan

**Keywords:** enhanced recovery after surgery, Smartband, minimal invasive surgery, colorectal surgery, quality of recovery

## Abstract

**Background:** Enhanced recovery after surgery (ERAS) is valuable in perioperative care for its ability to improve short-term surgical outcomes and facilitate patient recuperation after major surgery. Early postoperative mobilization is a vital component of the integrated care pathway and is a factor strongly associated with successful outcomes. However, early mobilization still has various definitions and lacks specific strategies.

**Methods:** Patients who underwent minimally invasive surgery for colorectal cancer followed our perioperative ERAS program, including mobilization from the first postoperative day. After perioperative care skills were improved in our well-established program, compliance, inpatient surgical outcomes, and complications associated with adding smartband use were evaluated and compared with the outcomes for standard protocol. Quality of recovery was evaluated using patient-rated QoR-40 questionnaires the day before surgery, on postoperative days 1 and 3, and on the day of discharge.

**Results:** Smartband use after minimally invasive colorectal surgery failed to increase compliance with early mobilization or reduce the occurrence of postoperative complications significantly compared with standard ERAS protocol. However, when smartbands were utilized, quality of recovery was optimized and patients returned to their preoperative status earlier, at postoperative day 3. The length of hospital stay, as defined by discharge criteria, and hospital stay of patients without complications was reduced by 1.1 and 0.9 days, respectively (*P* = 0.009 and 0.049, respectively).

**Conclusions:** Smartbands enable enhanced communication between patients and surgical teams and strengthen self-management in patients undergoing minimally invasive colorectal resection surgery. Accelerated recovery to preoperative functional status can be facilitated by integrating smartbands into the process of early mobilization during ERAS.

## Introduction

For both malignant and benign diseases, the most common postoperative complications after colorectal surgery are prolonged ileus, pneumonia, difficulty weaning from ventilation, and urinary tract infection. Minimally invasive surgery (MIS) improves some short-term surgical outcomes (fewer wound infections and lower wound dehiscence rate) and long-term outcomes (fewer early and late postoperative bowel obstructions). However, the typical length of hospital stay (LOS) after major colorectal surgery still varies.

Since the 1990s, alongside the development of regional anesthetic techniques and the widespread use of minimally invasive laparoscopic techniques, the concept of enhanced recovery after surgery (ERAS) has also become increasingly valued. The original ERAS study group was established in 2001 to improve patient recovery after major operations (1). In 2010, the ERAS society was founded in Sweden; thereafter, the guidelines for an integrated care pathway for colonic and rectal resection were outlined in 2012. These combined a range of simple evidence-based interventions aimed at improving postoperative recovery for patients after major colorectal surgery. Early mobilization is one intervention that is significantly associated with successful ERAS outcomes (2). Although no randomized control trial has supported the direct benefits of postoperative mobilization, prolonged immobilization increases the risk of pneumonia, insulin resistance, and muscle weakness (3). However, the definition of early mobilization varies, and specific strategies for it are lacking. Furthermore, objective assessment of the autonomy and involvement of patients is challenging. Discrepancy may exist between instruction and actual practice, which may impair the effectiveness of early mobilization and the quality of recovery.

Wearable devices are popular and commonly used in outdoor sports. They are also increasingly being used in medical contexts. For example, the use of step counters in patients with obstructive lung disease increases their physical activity and exercise capacity (4). The use and feasibility of wearable activity trackers to monitor and enhance postoperative early mobilization was also evaluated in a trial involving patients who underwent major visceral surgery (5). The aim of this study was to evaluate whether smartbands, which are popular wearable devices, help ERAS programs and improve recovery in patients who have undergone MIS for colorectal resection.

## Materials and Methods

This prospective non-randomized study included patients who had undergone MIS for colorectal cancer in a tertiary medical center by the same colorectal surgical team. Patients were excluded if they were aged <18 or >80 years, had an American Society of Anesthesiologist classification of ≥4, could not fully understand or follow instructions, had difficulty completing the required questionnaire, had received emergency operations, or were admitted to the intensive care unit after operation. This study was approved by the institutional review board of our hospital (KMUHIRB-F(II)-20180098). Informed consent was obtained from each patient before the integrated care pathway was carried out. All of the patients followed our perioperative ERAS protocol for elective colonic or rectal surgery, as recommended by the ERAS society (3, 6). At least two members of our surgical team evaluated each patient's discharge safety. Patients were discharged when they met all the associated criteria (adequate pain control with non-opioid oral analgesic medication, absence of fever, adequate oral intake, passage of stool, and same level of mobility as their preoperative status) and accepted the discharge. To adjust for the potential impact of non-medical variables (e.g., social problems and nursing home waiting list) on LOS, a LOS defined by discharge criteria (LOS-DC)—LOS until all the predefined discharge criteria were met regardless of acceptance of discharge by the patients—was also assessed (7).

**Figure 1:**
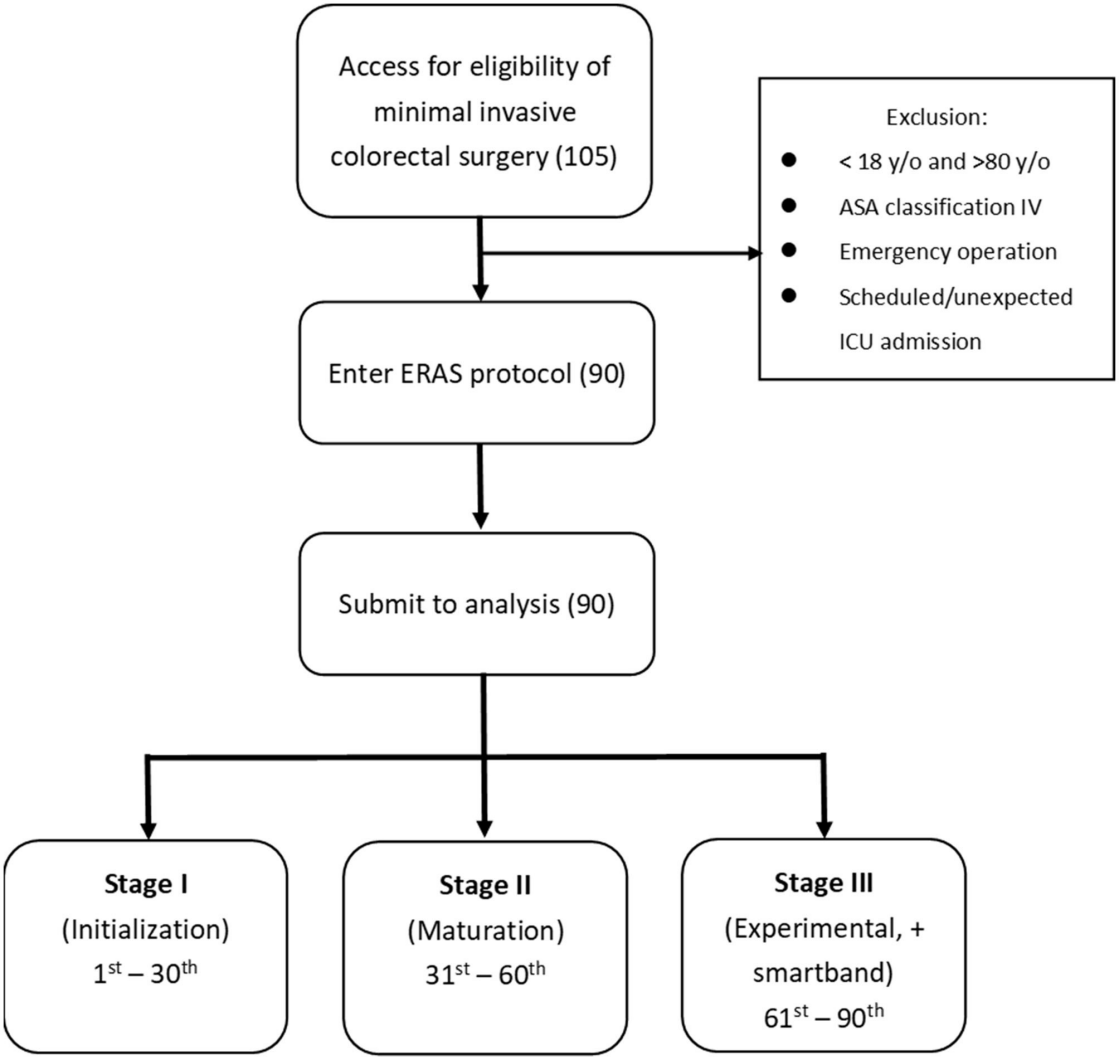
Consort diagram.

All patients whose data underwent analysis were allocated serial numbers and were grouped into three sequential stages: patients 1–30 formed the initialization stage (stage I), 31–60 formed the maturation stage (stage II), and 61–90 formed the experimental stage (stage III) and were assigned smartbands (Xiaomi Mi 2®, Xiaomi Corp., China) every day postoperatively (Figure 1). Stages II and III indicated that the perioperative care skill was mature and that the ERAS program was well-established, whereas stage I indicated that the program was initially operated and the skill was still raw. The primary endpoint of this study was compliance with the ERAS program, especially early mobilization. The secondary endpoints were short-term surgical outcomes and the quality of recovery. Patients' characteristics, compliance with each protocol item, and short-term surgical outcomes (including LOS, time to recovery of bowel function, time to resuming oral intake, 30-day complications, and readmission) were compared for each stage. The quality of recovery was compared between stages II and III.

### ERAS Protocol of Our Practice

- Detailed preoperative information, education, and counseling should be provided.
- Patients should abstain from smoking for the 24 h preceding surgery.
- Routine mechanical bowel preparation (MBP) should not be applied for right-side colonic surgery; oral antibiotics should be combined with MBP in left-side colonic surgery.
- Routine prophylaxis with intravenous antibiotics should be administered 30–60 min before colorectal surgery.
- A standard anesthetic protocol for rapid awakening should be used.
- Normothermia should be maintained intraoperatively.
- Fentanyl (a short-acting opioid) patient-controlled analgesia (PCA) is recommended. If intravenous opioids are used, the dose should be titrated to minimize the risk of side effects.
- A multimodal approach to postoperative nausea and vomiting prophylaxis should be adopted for all patients.
- Postoperative nasogastric (NG) tubes should not be used routinely. NG tubes inserted during surgery should be removed before anesthesia reversal.
- Transurethral bladder drainage within 1 day after colonic surgery and 3 days after rectal surgery is recommended.
- Postoperative ileus should be prevented (e.g., by administering postoperative laxatives).
- Postoperative multimodal analgesia should be used to limit the use of opioids.
- Postoperative early enteral feeding should be undertaken (i.e., clear liquid diet within 24 h and full diet within 48 h).
- Intravenous fluids should be discontinued as soon as is practicable.
- Early mobilization should be encouraged.

### Smartbands

All participants were instructed to be active from postoperative day one (POD1). Standard care dictated at least 30 min of out-of-bed activity at POD1 and 1 h thereafter. Patients in stage III wore Xiaomi Mi bands on their wrists postoperatively. The cumulative number of steps taken, walking time, and walking distance were displayed on the screen and could easily be read by patients or their caregivers. Our team recorded the parameters daily using smartphones and apps (Mi Band App).

### Quality of Recovery Score

The quality of recovery score (QoR-40) is a recovery-specific and patient-rated questionnaire containing 40 items measuring five dimensions: physical comfort (12 items), emotional state (nine items), physical independence (5 items), psychological support (7 items), and pain (7 items). The QoR-40 was originally developed and validated in Australia in 2000 (8). The total score and those for subscales of the QoR-40 are measured using a 5-point Likert scale (for positive items: 1 = *none of the time*, and 5 = *all of the time*; for negative items, the scoring is reversed). Individual scores are then summed, with the minimum and maximum scores being 40 and 200 points, respectively. The QoR-40 has also been validated in East Asian countries for evaluating the quality of recovery after surgery and the quality of anesthesia methods (9, 10). Patients in our study completed the questionnaire the day before operation (baseline), on POD1, on POD3, and on the day of discharge.

### Statistical Analysis

Qualitative variables are expressed as numbers and percentages, and quantitative variables are expressed as the median and standard deviation (SD). Comparisons between stages were performed using the χ^2^ test for categorical variables and the F-test for quantitative variables. Tukey's honest significance test was used for *post hoc* testing between each pair of stages. For comparison of QoR-40 test scores, Student's *t* test was used to evaluate changes between measurements. The α error was set at 0.05. Statistical analysis was performed using JMP 13.0.0 (SAS Institute, Cary, NC, USA) for Windows. A *P* < 0.05 was considered statistically significant.

## Results

### Compliance With ERAS Components

Data were collected from May 2017 to February 2019. A total of 105 patients were enrolled for evaluation of eligibility, and 90 patients entered the ERAS protocol and were ultimately analyzed (Table 1). Charlson index scores were compared, and no potential confounding effect of preexisting medical comorbidities were noted between stages. Early mobilization was observed in 20 (66.7%) patients in stage I, and this proportion increased to 80% in stage II. Of the patients in stage III wearing smartbands, 25 (83.3%) achieved early mobilization. Compliance with respect to early mobilization was not significantly increased in patients with smartbands compared with those without smartbands (Table 2).

**Table 1:** Patient demographic data at each stage.

	**Stage I (initialization stage)**	**Stage II (maturation stage)**	**Stage III (experimental stage)**	***P***
Age (SD)	57.6 (10.4)	61.6 (6.8)	59.8 (9.1)	0.217
Male (%)	16 (53.3)	14 (46.7)	19 (63.3)	0.424
BMI (SD)	25.4 (4.4)	24.3 (2.7)	24.0 (4.0)	0.313
ASA 1/2/3 (%)	2/23/5 (6.7/76.7/16.7)	0/20/10 (0/66.7/33.3)	0/21/9 (0/70.0/30.0)	0.165
CCI (SD)	4.6 (1.9)	4.4 (1.6)	4.3 (1.5)	0.870
Robot/Laparoscopic (%)	19/11 (63.3/36.7)	26/4 (83.7/13.3)	23/7 (76.7/23.3)	0.104
RH/AR/LAR (%)	4/5/21 (13.3/16.7/70.0)	3/4/23 (10.0/13.3/76.7)	4/6/20 (13.3/20.0/66.7)	0.937
CEA, ng/mL (SD)	2.51 (2.04)	2.65 (2.28)	2.34 (1.69)	0.851
Albumin, mg% (SD)	4.36 (0.28)	4.36 (0.31)	4.39 (0.29)	0.871
CCRT (%)	15 (50)	18 (60.0)	17 (56.7)	0.730

*BMI, body mass index; ASA, American society of anesthesiologists; CCI, Charlson comorbidity index; RH, right hemicolectomy; AR, anterior resection; LAR, low anterior resection; CEA, carcinoembryonic antigen; CCRT, concurrent chemoradiotherapy*.

**Table 2:** Compliance with ERAS components at each stage.

**ERAS items**	**Stage I**	**Stage II**	**Stage III**	***P***
No smoking/cessation (%)	30 (100)	30 (100)	30 (100)	-
No MBP in right side colonic surgery / MBP + OA in left side colonic surgery (%)	30 (100)	30 (100)	29 (96.7)	0.330
Prophylaxis antibiotics (%)	30 (100)	30 (100)	29 (96.7)	0.330
PCA (%)	10 (33.3)	13 (43.3)	20 (66.7)	0.028^*^
No additional opioid (%)	21 (70.0)	28 (93.3)	26 (86.7)	0.045^*^
Ileus prevention (%)	28 (93.3)	29 (96.7)	26 (86.7)	0.338
PONV prophylaxis (%)	29 (96.7)	25 (83.3)	29 (96.7)	0.099
Early mobilization (%)	20 (66.7)	24 (80)	25 (83.3)	0.281
Remove Foley as schedule (%)	27 (90)	29 (96.7)	27 (90.0)	0.441
No NG / Remove NG before reversal of anesthesia (%)	23 (76.7)	24 (80.0)	29 (96.7)	0.041^*^
Clear liquid diet in 24 h after operation (%)	29 (96.7)	29 (96.7)	29 (96.7)	1.000
Full diet in 48 h after operation (%)	28 (93.3)	28 (93.3)	26 (86.7)	0.594

*MBP, mechanical bowel preparation; OA, oral antibiotics; PCA, patient-controlled analgesia; PONV, postoperative nausea and vomiting; NG, nasogastric*.

* *p < 0.05*.

All patients but one consumed a clear liquid diet for 24 h before surgery and took commercial Bowklean® powder suspension (magnesium oxide, sodium picosulfate, and citric acid anhydrous; Genovate Biotechnology, Taiwan) in split doses for MBP. Patients also took oral metronidazole and neomycin for MBP. PCA with intravenous fentanyl was used in 10, 13, and 20 (33.3, 43.3, and 66.7%) patients in stage I, II, and III, respectively, for 3 days (range 2–4 days) postoperatively. Widespread use of PCA was discouraged mostly due to the personal considerations of patients. Multimodal analgesia was applied to all patients to avoid excessive opioid use. However, 9 (30%), 2 (6.7%), and 4 (13.3%) patients in stages I, II, and III, respectively, received additional opioid treatment postoperatively for pain relief. Significantly lower proportions of patients received additional opioid treatment in stages II and III compared with the proportion of patients receiving opioid treatment in stage I (*P* = 0.045). Removal of transurethral bladder drainage 1 day after colonic surgery and 3 days after rectal surgery was recommended. This was achieved in at least 90% of patients in each stage, and only one episode of urinary retention occurred. The prohibition of routine NG tube use was adhered to. If decompression was indicated, the NG tubes were removed before reversal of anesthesia. This was undertaken in 23, 24, and 29 (76.7, 80, and 96.7%) patients in stages I, II, and III, respectively, and compliance was significantly improved (*P* = 0.041) after the implementation of ERAS. In terms of diet, 87 of 90 (96.7%) patients resumed early enteral feeding, starting with a clear liquid diet 24 h after operation. The goal of a full diet within 48 h postoperatively was achieved for 86.7–93.3% of patients. As our skill with the ERAS protocol improved so did patient compliance; patient compliance was ≥80% for all items in stages II and III, except for the PCA ratio.

### Short-Term Surgical Outcomes

The mean total LOS in stage I was 11.4 days (SD = 2.8 days), which significantly decreased to 10.1 days (SD = 1.0 days; *P* = 0.023) in stage II and to 10.0 days (SD = 1.3 days; *P* = 0.016) in stage III (Table 3). The total LOS in stage III and in stage II was almost identical (*P* = 0.990). After cases with complications were excluded, the LOS in uncomplicated patients decreased from 10.7 days (SD = 2.1 days) in stage I to 10.0 days (SD = 0.9 days) in stage II, without statistical significance (*P* = 0.180). However, the LOS of uncomplicated patients wearing smartbands in stage III exhibited a further decrease to 9.8 days (SD = 0.7 days) compared with stage I (*P* = 0.049). LOS-DC was also significantly reduced—from 8.9 days (SD = 1.6 days) in stage I to 7.8 days (SD = 1.4 days) in stage III (*P* = 0.009). However, LOS-DC in stage II (8.5 days, SD = 1.0 days) was not significantly shorter than that in stage I (*P* = 0.440). The time to recovery of bowel function, including time to flatus passage and time to stool passage, was not shortened after implementation of the ERAS protocol or after introduction of smartbands during perioperative care. Patients started to drink clear liquid one day after MIS in stages I and II. A full diet was resumed 2.6 days (SD = 1.4 days) postoperatively in stage I and 2.1 days (SD = 0.6 days; *P* = 0.127) in stage II. Patients in stage III had similar outcomes in early enteral feeding compared with those in stage II.

**Table 3:** Short-term surgical outcomes and complications at each stage.

**Outcome measurement**	**Stage I**	**Stage II**	**Stage III**	**Stage II vs. stage I**	**Stage III vs. stage II**	**Stage III vs. stage I**
Total LOS, days (SD)	11.4 (2.8)	10.1 (1.0)	10.0 (1.3)	*P* = 0.023^*^	*P* = 0.990	*P* = 0.016^*^
Total LOS, uncomplicated, days (SD)	10.7 (2.1)	10.0 (0.9)	9.8 (0.7)	*P* = 0.180	*P* = 0.821	*P* = 0.049^*^
LOS-DC, days (SD)	8.9 (1.6)	8.5 (1.0)	7.8 (1.4)	*P* = 0.440	*P* = 0.177	*P* = 0.009^*^
Clear liquid diet since, days (SD)	1.0 (0.4)	1.0 (0.4)	1.1 (0.3)	*P* = 1.000	*P* = 0.547	*P* = 0.547
Full diet since, days (SD)	2.6 (1.4)	2.1 (0.6)	2.2 (0.6)	*P* = 0.127	*P* = 0.841	*P* = 0.343
Flatus passage since, days (SD)	1.5 (0.6)	1.5 (0.8)	1.6 (0.9)	*P* = 0.984	*P* = 0.861	*P* = 0.767
Stool passage since, days (SD)	2.1 (1.0)	2.2 (0.9)	2.4 (1.0)	*P* = 0.859	*P* = 0.788	*P* = 0.465
Medical complications (%)	4 (13.3)	3 (10.0)	2 (6.7)	*P* = 0.686		
Surgical complications (%)	1 (3.3)	0 (0)	1 (3.3)	*P* = 0.439		
Total complications (%)	4 (13.3)	3 (10.0)	2 (6.7)	*P* = 0.686		
Reoperation (%)	1 (3.3)	0 (0)	1 (3.3)	*P* = 0.439		
Readmission (%)	0 (0)	1 (3.3)	0 (0)	*P* = 0.330		
Mortality (%)	0 (0)	0 (0)	0 (0)	-		

*LOS, length of (hospital) stay; LOS-DC, length of hospital stay defined by discharge criteria*.

* *p < 0.05*.

### Complications

The number of complications in each stage in our study tended to decrease after implementation of ERAS. The total complication rate was 13.3, 10.0, and 6.7% in stages I, II, and III, respectively (*P* = 0.686). In stage I, four cases had complications. Symptomatic anastomotic leakage was observed 6 days after robotic LAR in a 46-year-old male patient, and he developed deep surgical site infection and sepsis with a total LOS of 19 days. Another three patients developed chylous ascites with ileus, urinary tract infection, and pneumonia, respectively. In stage II, three medical complications were observed. A 60-year-old female patient who received concurrent chemoradiotherapy and robotic LAR exhibited intraabdominal infection and ileus after being discharged from her ward (postoperative LOS, 6 days). She was readmitted for antibiotic treatment. Surgical site infection and prolonged postoperative ileus developed in a 61-year-old male patient with diabetes. Acute urinary retention is another example of complications occurring in stage II. A 59-year-old female patient with a BMI of 30 who received laparoscopic RH in stage III developed anastomotic leakage and severe intraabdominal infection. She underwent reoperation, and the total LOS was 16 days. A patient in stage III also experienced prolonged ileus.

### Quality of Recovery Score

**Figure 2:**
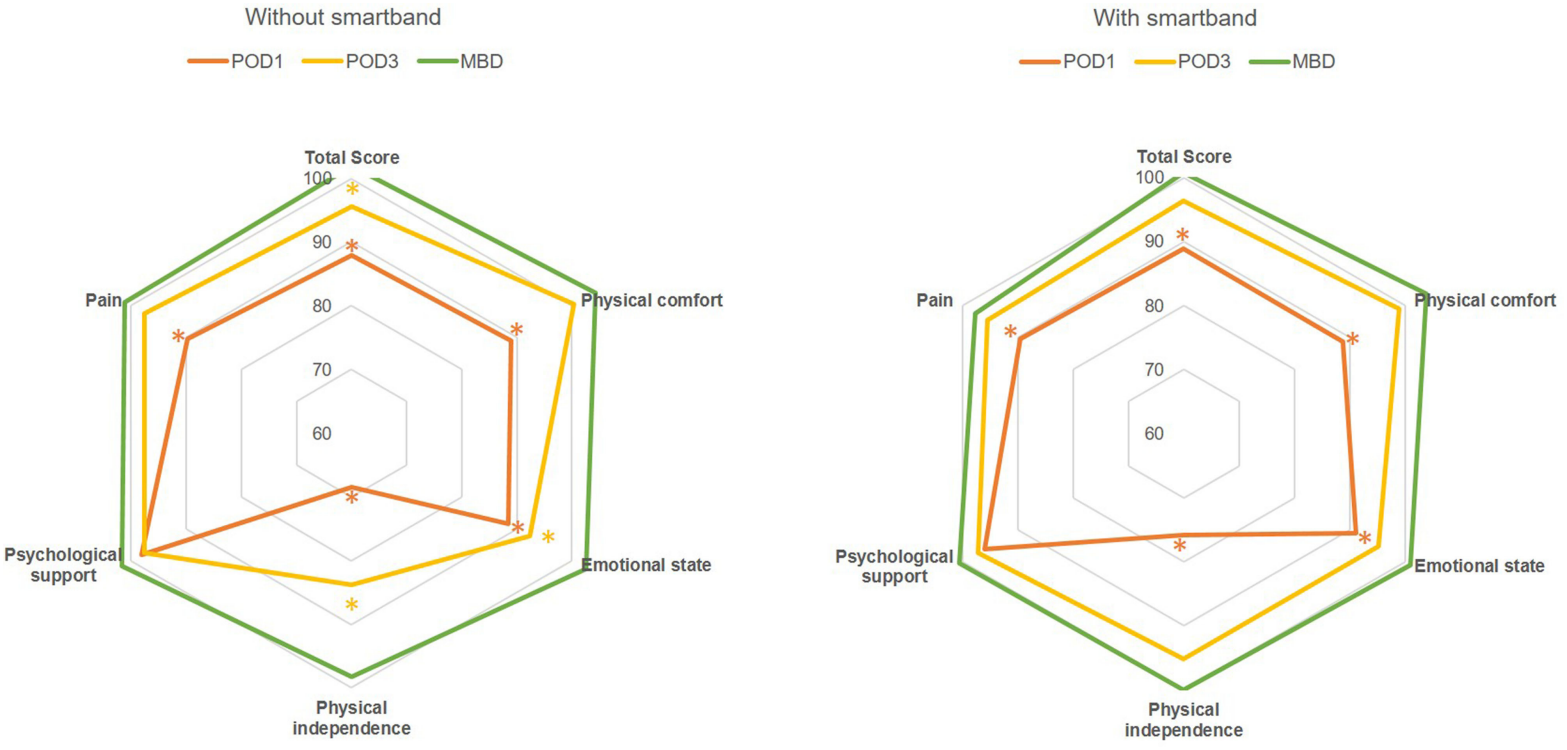
Rader chart of total score and scores for subscales (physical comfort, emotional state, physical independence, psychological support, and pain) of QoR-40 with and without smartband integration in a perioperative ERAS program following minimally invasive colorectal surgery. The grid lines mark the percentage of each dimensional score compared with the preoperative measurement. Asterisks: significant difference (*P* < 0.05) compared with preoperative functional status; POD, postoperative day; MBD, may be discharged.

To evaluate the quality of recovery after surgery and anesthesia, QoR-40 was used to measure each patient's health status preoperatively at POD1, at POD3, and on the day of discharge. Scores were compared only between stages II and III, when the enhanced recovery program had been well-established and ERAS skills were enhanced in our surgical team. In the stage II group, the preoperative total baseline score was 178.3 points, and this decreased to a minimum of 156.8 points at POD1 (−21.5 points, *P* < 0.0001; Table 4 and Figure 2). All dimensions decreased synchronously except for psychological support. The total score recovered to 170.4 points (−7.9 points) by POD3, but this was still significantly lower than the baseline score (*P* = 0.046). The scores for emotional state (35.1 points, −2.9, *P* = 0.037) and physical independence (19.6 points, −3.8, *P* < 0.0001) remained much lower than they had been preoperatively. Scores in all dimensions returned to or were better than baseline scores by the day of discharge. The total score was 181.9 points (plus 3.6, *P* = 0.369) at the day of discharge in the stage II group.

**Table 4:** QoR-40 score (total and subscale) in patients with and without postoperative smartband use.

**STAGE II (WITHOUT SMARTBAND)**										
	**Preoperative**	**POD1**	**POD3**	**MBD**	**POD1-Preoperative**	***P***	**POD3-Preoperative**	***P***	**MBD-Preoperative**	***P***
Physical comfort (60)	52.2	46.4	52.4	54.4	−5.8	<0.001^*^	+0.2	0.872	+2.2	0.084
Emotional state (45)	38	33.6	35.1	39	−4.4	0.002^*^	−2.9	0.037^*^	+1.1	0.446
Physical independence (25)	23.4	16	19.6	23	−7.4	<0.0001^*^	−3.8	<0.0001^*^	−0.4	0.612
Psychological support (35)	31.8	31.2	31.2	32.3	−0.6	0.517	−0.7	0.483	+0.4	0.666
Pain (35)	32.9	29.5	32.1	33.3	−3.4	<0.0001^*^	−0.8	0.126	+0.3	0.533
Total (200)	178.3	156.8	170.4	181.9	−21.5	<0.0001^*^	−7.9	0.046^*^	+3.6	0.369
**STAGE III (WITH SMARTBAND)**										
	**Preoperative**	**POD1**	**POD3**	**MBD**	**POD1-Preoperative**	***P***	**POD3-Preoperative**	***P***	**MBD-Preoperative**	***P***
Physical comfort (60)	53.3	47.3	52.7	55.3	−6.0	0.0002^*^	−0.6	0.689	+2.0	0.216
Emotional state (45)	39.4	35.9	37.5	39.8	−3.5	0.013^*^	−1.9	0.175	+0.4	0.760
Physical independence (25)	23.1	17.5	22.0	23.1	−5.6	<0.0001^*^	−1.2	0.182	−0.1	0.953
Psychological support (35)	32.5	31.2	31.6	32.7	−1.3	0.138	−0.9	0.282	+0.2	0.822
Pain (35)	33.4	29.9	31.9	32.6	−3.5	<0.0001^*^	−1.5	0.055	−0.7	0.355
Total (200)	182.1	161.9	175.4	183.5	−20.2	<0.0001^*^	−6.8	0.099	+1.4	0.741

*POD, postoperative day; MBD, may be discharged*.

* *p < 0.05*.

QoR-40 score was also recorded in stage III, when patients were assigned to wear smartbands postoperatively. The total preoperative score was 182.1 points; similarly, this significantly declined to a minimum of 161.9 (−20.2, *P* < 0.0001) by POD1. At POD3, the total score and scores for all 5 dimensions recovered to the baseline level. The scores for emotional state (37.5 points, −1.9, *P* = 0.175) and physical independence (22.0 points, −1.2, *P* = 0.182) in POD3 seemed to improve more rapidly postoperatively after the introduction of smartbands into ERAS perioperative care. Compared with the total QoR-40 score of patients in stage II, that of patients in stage III recovered to an adequate level by POD3 (175.4 points, −6.8, *P* = 0.099). The average score on the day of discharge was 183.5 points, 1.4 points higher than the baseline score (*P* = 0.741), with all dimensions exhibiting recovery to baseline of higher scores.

## Discussion

Wearable devices have wide-ranging clinical applications, including cardiopulmonary and vascular monitoring, glucose monitoring, neurological function monitoring, physical therapy, and rehabilitation. Smartbands augment the physician–patient relationship, increase the autonomy and involvement of patients with respect to their health care, and enable the application of novel remote monitoring techniques (11). Use of wearable devices in the management of osteoarthritis leads to psychosocial effects: clinician–patient communication improves, and patients are empowered to improve self-management (12).

In the current study, the integration of smartbands into a perioperative ERAS program did not significantly increase patient compliance in terms of early mobilization. It might affect short-term surgical outcomes and quality of recovery by means of parameters other than compliance of early mobilization. Through exchangeable and objective information, smartbands ostensibly enable more effective communication between patients and surgical teams, and they enhance self-management in patients receiving MIS for colorectal resection. Earlier return to preoperative functional status, especially with respect to emotional state and physical independence, was facilitated at POD3 through postoperative smartband use (Figure 2, the total score and scores for subscales of emotional state and physical independence recovered to an adequate level by POD3 after the introduction of smartbands into ERAS perioperative care). LOS in patients without complications and LOS-DC decreased after the introduction of smartbands in our well-established ERAS program. Furthermore, although the effective LOS did not decrease in this study, this is reasonably anticipated in the near future.

ERAS is composed of 10 to more than 20 elements. These perioperative care measures are usually divided into three phases: preoperative, intraoperative, and postoperative. Only a few involve the surgical procedure itself, but several diverge from conventional perioperative care. Several meta-analyses and systemic reviews have compared the ERAS program to traditional care for colorectal surgery (13). Zhuang et al. reviewed 13 randomized control trials with at least seven documented ERAS elements and concluded that ERAS reduced LOS (by 2–3 days) without increasing the readmission rate. It also reduced the occurrence of general and medical complications. Mortality rate was not higher than that observed in conventional care, and bowel function recovery was faster (by 1 day) with ERAS (14).

The introduction of the ERAS protocol in perioperative care for laparoscopic colorectal surgery is a gradual process. The average compliance improves the longer an ERAS protocol has been active. LOS is inversely correlated with compliance. Pedziwiatr et al. reported that at least 30 patients and a period of 6 months were required to meet an average compliance level of 80% (15). This conclusion informed the basic design of our study.

With consideration of health economics and quality of life, ERAS can be recommended because it is likely to reduce costs and improve the quality of recovery. To assess the quality of life after surgery for colorectal cancer, King et al. used the European Organization for Research and Treatment of Cancer core quality of life questionnaire (colorectal module), a valid measure that has been used in cancer patients. The role function score, physical functional score, pain score, and fatigue score tended to demonstrate more favorable results in enhanced recovery programs than in conventional care (16).

Early mobilization is a crucial ERAS element in almost all fields. It reduces chest complications and counteracts insulin resistance (17). The combination of mobilization and nutritional support results in improved muscle strength after colorectal surgery (18). Following laparoscopic colorectal surgery, a lack of early mobilization is significantly associated with prolonged hospital stay (19). However, appropriate ambulation goals (e.g., for steps, distance, or duration) for early mobilization are undefined. The question remains whether specific strategies (e.g., accompanied walks or supervised exercise) benefit patients receiving minimally invasive colorectal surgery. Wiklund et al. observed that goals for steps did not significantly improve bowel function recovery or shorten LOS in patients undergoing gastric-bypass surgery (20). Staff-assisted facilitation of early mobilization also did not improve outcomes compared with traditional ERAS care for colorectal surgery (21).

To the best of our knowledge (according to a search of PubMed websites until March 2020), this is the first study aiming to evaluate the effectiveness of integrating smartbands into a perioperative ERAS program after MIS for colorectal resection. Nonetheless, this study had some limitations, including the non-randomized design and the insufficient representation of patients enrolled. Moreover, the impact of improving compliance with other ERAS elements (e.g., proportion of PCA use and postoperative NG tube indwelling) on short-term surgical outcomes and the quality of recovery in our patients was not thoroughly analyzed. The actual efficacy and extended applications of smartbands in ERAS should be investigated in the future.

## Conclusions

Smartbands enhance communication between patients and surgical teams and strengthen self-management in patients undergoing MIS for colorectal resection. Accelerated recovery to preoperative functional status can be facilitated by integrating smartbands into the process of early mobilization in a well-established ERAS program. Further reductions in effective LOS are reasonably anticipated.

## Data Availability Statement

The raw data supporting the conclusions of this article will be made available by the authors, without undue reservation.

## Ethics Statement

The studies involving human participants were reviewed and approved by Institutional Review Board, Kaohsiung Medical University Hospital (KMUHIRB-F(II)-20180098). The patients/participants provided their written informed consent to participate in this study.

## Author Contributions

T-CY, being the first author of this manuscript, designed this study, analyzed the data, and wrote the manuscript. C-WH, H-LT, W-CS, C-JM, and T-KC made substantial contributions in terms of the data acquisition, interpretation, and statistical analyses, in addition to assisting with the manuscript preparation. J-YW, being the corresponding author for this manuscript, also participated in the study design and coordination, in addition to making critical revisions to the manuscript. All have reviewed and approved submission of the final version of the manuscript.

## Conflict of Interest

The authors declare that the research was conducted in the absence of any commercial or financial relationships that could be construed as a potential conflict of interest.
